# Supplementary material for: Relative importance of informational items in participant information leaflets for trials: a Q-methodology approach
Source: BMJ Open. 2018 Sep 5;8(9):e023303. doi: 10.1136/bmjopen-2018-023303 (PMC6129101; doi:10.1136/bmjopen-2018-023303)

Additional File 1. The vignette used in the Q-sort

Potential trial participants

Imagine you are in a consultation with your doctor. The doctor is discussing with you what treatment you could have for your chronic condition. You are suitable to take part in a clinical trial run by the NHS. If you decide to take part, you will be randomly allocated to either treatment A or B.

What information would be important to you when making the decision to take part?

Research nurses

Imagine you are recruiting patients to a clinical trial, run by the NHS. The trial is comparing treatment A and treatment B for a chronic condition, and those who agree to take part are randomly allocated to either treatment A or treatment B.

What information would be important to potential participants when making the decision to take part?

Additional File 2. The 32-item Q-grid used for the Q-sort

Q grid

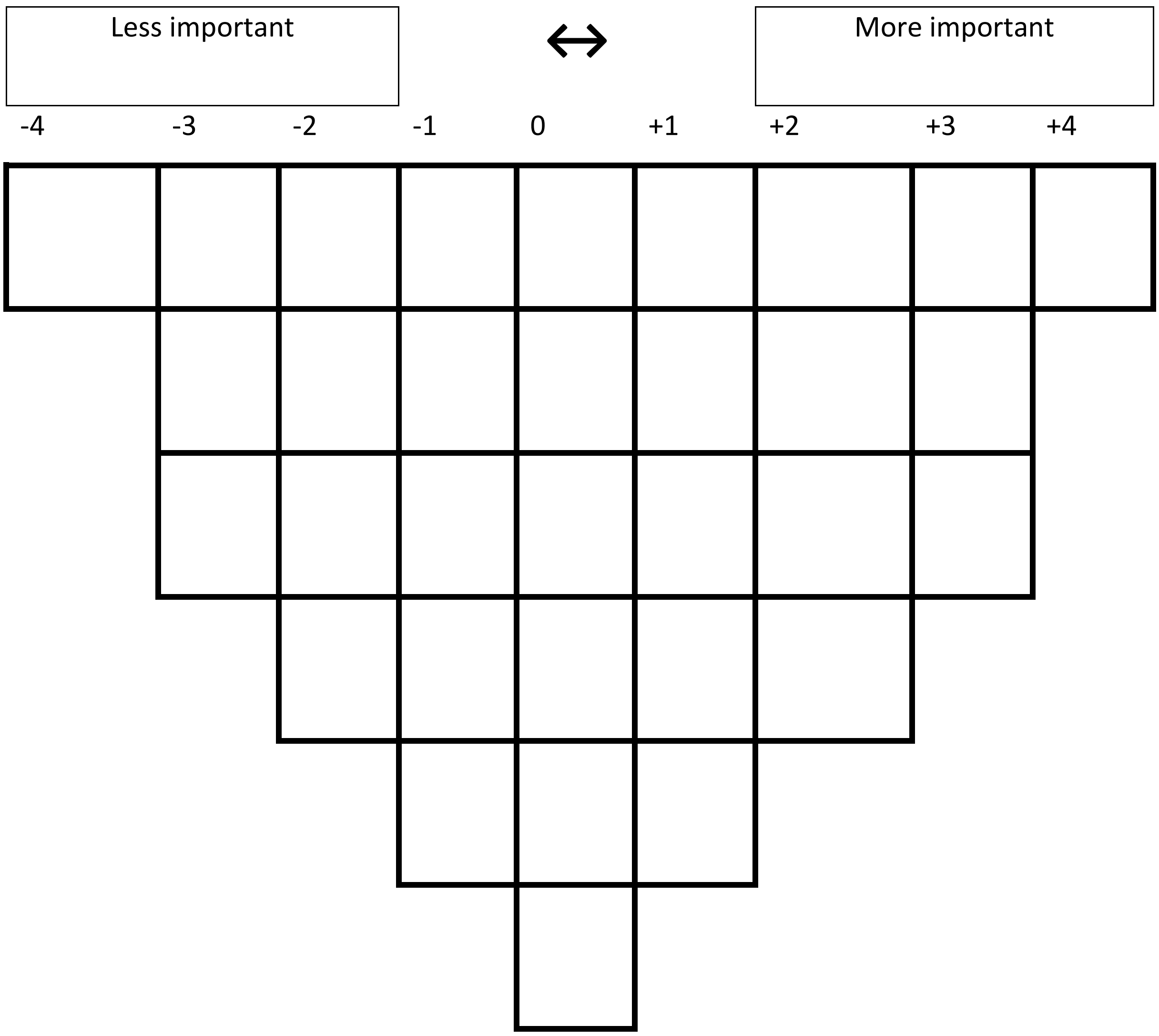

Supplement: Supplementary file 1 [file bmjopen-2018-023303supp001.pdf]
